# Draft genome of *Ochrobactrum intermedium* strain M86 isolated from non-ulcer dyspeptic individual from India

**DOI:** 10.1186/1757-4749-5-7

**Published:** 2013-04-04

**Authors:** Girish Kulkarni, Dhiraj Dhotre, Mahesh Dharne, Sudarshan Shetty, Somak Chowdhury, Vatsala Misra, Sriprakash Misra, Milind Patole, Yogesh Shouche

**Affiliations:** 1 Molecular Biology Unit, National Centre for Cell Science, Pune, India; 2 Microbial Culture Collection, National Centre for Cell Science, Pune, India; 3 NCIM Resource Centre, National Chemical Laboratory, Pune, India; 4 Department of Pathology, Moti Lal Nehru Medical College, University of Allahabad, Allahabad, India; 5 Department of Gastroenterology, Moti Lal Nehru Medical College, University of Allahabad, Allahabad, India

## Abstract

**Background:**

*Ochrobactrum intermedium* is an emerging opportunistic pathogen of humans that is closely related to members of the genus *Brucella*. Earlier, we reported the case of an Indian subject with non-ulcer dyspeptic symptoms whose urease positive gastric biopsy revealed the presence of *Helicobacter pylori* along with non-*Helicobacter* like bacteria, eventually cultured and identified as *O. intermedium* strain M86.

**Results:**

Here, we describe the unclosed draft genome of the strain M86 with a length of 5,188,688 bp and mean G+C content of 57.9%. We have also identified many putative gene clusters that might be responsible for its persistence in the gastric mucosa.Comparative analysis of genomic features of *Ochrobactrum intermedium* strain M86 and *Ochrobactrum intermedium* LMG 3301^T^ was also done.

**Conclusions:**

This paper attempts to gain whole-genome based insights into the putative gene determinants of *O. intermedium* for survival in the highly acidic stomach lumen environment .Identification of genes putatively involved in the various metabolic pathways may lead to a better understanding of the survival of *O. intermdedium* in acidic condition.

## Background

The *Alphaproteobacteria* represent a biologically diverse group of bacteria with members like *Brucella, Bartonella, Agrobacterium* and *Ochrobactrum* that are capable of interacting with eukaryotic cells. *Ochrobactrum intermedium* is a Gram-negative, capsulating, aerobic bacilli belonging to the *Alphaproteobacteria.* It is the closest genetic relative of genus *Brucella* as evidenced by protein profiling, western blot, immunoelectrophoresis, amplified fragment length polymorphism, 16S rRNA gene and *RecA* gene sequence based studies [1]. Previous reports have suggested *O. intermedium* as an emerging pathogen in liver abscess post-liver transplantation and in the bladder cancer patient causing presumptive bacteremia [2,3]. But, clinical manifestations and diseases caused by *O. intermedium* are poorly characterised.

Several bacteria other than *Helicobacter pylori* have been detected earlier in gastric biopsies. The presence of *O. intermedium* along with *H. pylori* was reported earlier in a subject from North-India diagnosed with non-ulcer dyspepsia [4]. A unique observation was the presence of severe fibrosis in the lamina propria of the gastric mucosa revealed during histological examination of the gastric antral biopsy. Whether this fibrosis was caused either partially or totally by *O. intermedium* was not clear. Other species of *Ochrobactrum* have also been associated along with *H. pylori,* e.g., *O. anthropi* with mild gastritis in squirrel monkeys [5]. Similarly, *Gastrospirillum hominis*[6], enterococcci [7] and staphylococci have been associated with gastric disorders [8]. In some cases, they have been isolated from antral biopsies from patients with or without *H. pylori* colonization [9].

Importantly, both *H. pylori* and *O. intermedium* produce urease, and thus the detection of *H. pylori* by urease test in the presence of *Ochrobactrum* may be confounded. The role of *Ochrobactrum* in gastric pathology remains uncertain and requires detailed pathologic, microbiological and genetic investigations in order to evaluate the link between *H. pylori* and *O. intermedium* in the gastric niche. This paper attempts to gain whole-genome based insights into the putative gene determinants of *O. intermedium* for survival in the highly acidic stomach lumen environment.

## Methods

### Genome sequencing

Genomic DNA was isolated by PureLink®Genomic DNA Kit. The draft genome sequence of strain M86 was determined by Ion Torrent Personal Genome Machine (PGM™) sequencer using a 316 chip with 200-bp single-end shotgun sequencing. A total of 2,602,696 reads were obtained. PGM sequencing resulted in about 67X genome coverage with 148 contigs.

### Assembly and annotation

The *de novo* approach was applied to finalize the unclosed draft genome using MIRA 3.4.0 version using default parameters [10]. Prediction and annotation of genes were done using RAST [11] server with SEED database and ISGA pipeline [12]. The data were further validated using gene prediction tools such as Glimmer [13]. Functional annotation was also performed by PGAAP using public database of National Centre for Biotechnology Information (NCBI). Prophages and putative phage like elements in the genome were identified using prophage-predicting PHAST [14] Web server. Regions identified algorithmically as “intact” by PHAST, as well as regions sharing a high degree of sequence similarity and conserved synteny with predicted “intact” prophages, were identified as prophages.

### Submission of genome sequence

The *Ochrobactrum intermedium* strain M86 whole genome shotgun (WGS) project was submitted to the GenBank and has the project accession AOGE00000000 and consists of sequences AOGE01000001-AOGE01000148

## Quality assurance

The genomic DNA was isolated from pure bacterial isolate and was further confirmed with 16S rRNA gene sequencing. Bioinformatic assessment of potential contamination of the genomic library by allochthonous microorganisms was done using PGAAP and RAST annotation systems.

## Initial findings

### Genome characteristics

**Figure 1:**
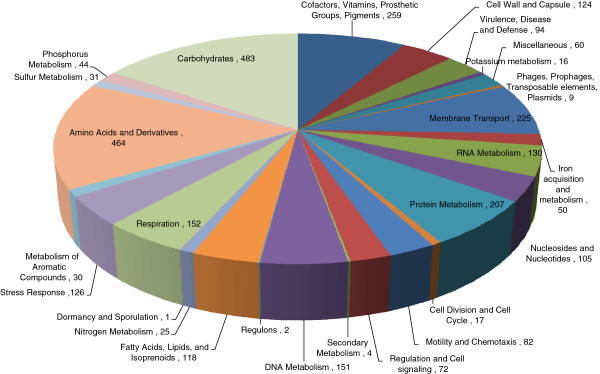
**Subsystem distribution statistic of *Ochrobactrum intermedium *strain M86 based on genome annotation performed according to RAST server.**

Genome of *O. intermedium* strain M86 was sequenced on the IonTorrent Personal Genome Machine (PGM™) using 316 chip that resulted in 2,602,696 total reads with a mean read length of 155 bp. *de-novo* assembly using the MIRA assembler v3.4.0 [10] with default parameters yielded ~67X coverage. A total of 148 contigs with >500 bp length were obtained. The unclosed draft genome sequence of strain M86 is of 5,188,688 bps and 5043 predicted coding DNA sequences (CDSs) and 66 RNA genes with mean G + C content of 57.9%. RAST server based annotation of the whole genome, showed the presence of 437 subsystems (related functional roles) [11]. Figure 1 describes the subsystem distribution of strain M86.

### Putative gene clusters responsible for survival of *Ochrobactrum intermedium* strain M86 in the acidic environment of stomach lumen

**Figure 2:**
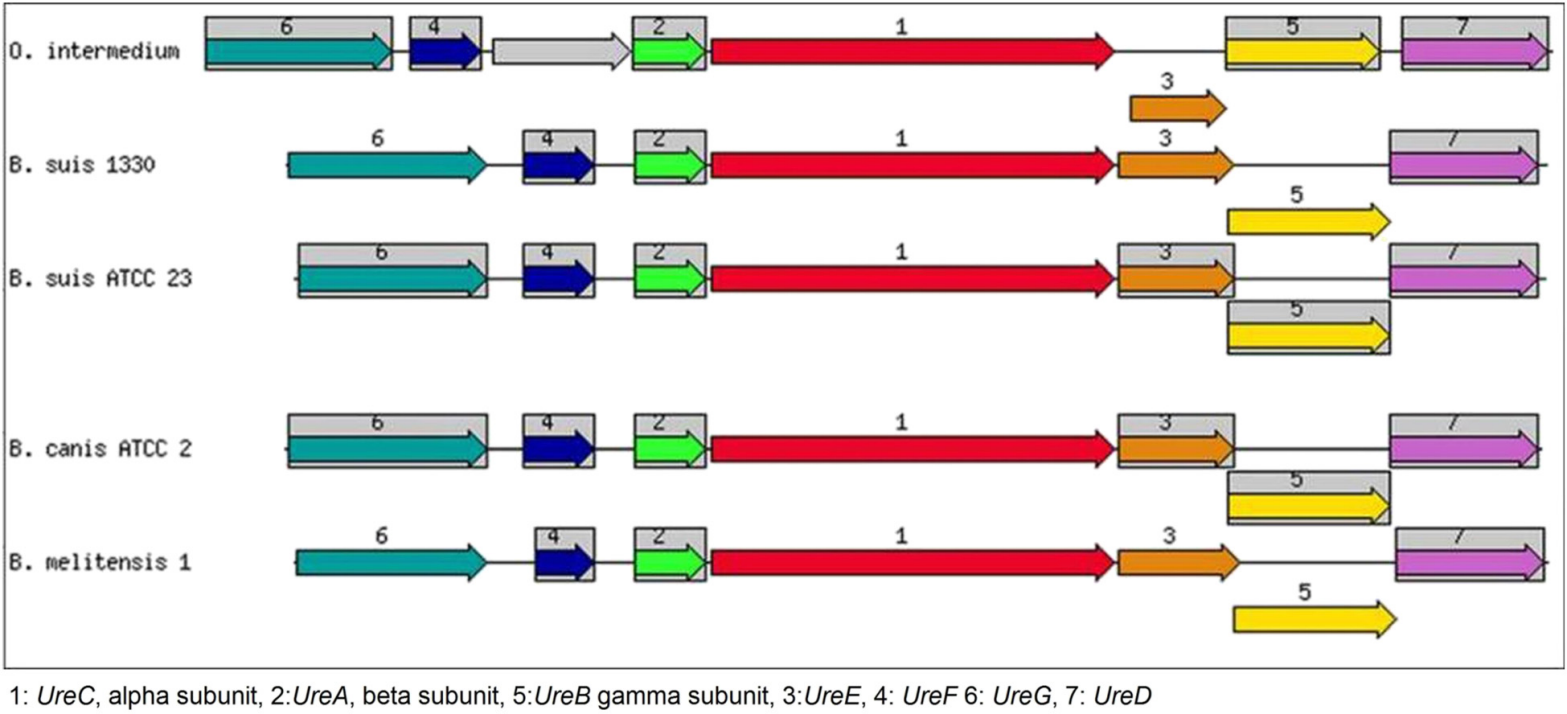
Genetic relatedness of urease gene cluster with closely related bacteria.

*H. pylori* have several genes for biosynthesis of cytosolic urease for its survival in the acidic environment of stomach lumen [15]. Genome of strain M86 contains urease gene cluster (see Figure 2): out of which, *UreA*, beta subunit, *UreB* gamma subunit, *UreC*, alpha subunit, are part of core Urease enzyme, While four accessory proteins: *UreD*, *UreE*, *UreF* and *UreG* play important role in Ni^2+^ uptake and insertion into active site of apo-enzyme. Genetic relatedness of urease gene cluster with phylogeneticaly closely related bacteria is shown in Figure 2. A complete operon encoding the *VirB* gene involved in conjugative transfer is present in strain M86. Genes encoding osmotic stress, oxidative stress *HPIIb*, cold shock *GrpE*, heat shock *DnaK*, periplasmic stress *DegQ* and protection from reactive oxygen species, *sod* are found. Genes predicated to encode flagellar biosynthesis protein *FlhA and FlhB* has been identified in genome of strain M86 which are likely elementary to adaptation of new lifestyle. Enterobactin synthesis clusters of *entA, entB1, entB2, entC, entD, entE, entF, entG, entH* genes were also observed in the genome sequence of strain M86 suggesting its ability of iron acquisition by siderophore production. Presence of membrane transport machinery with dominance of Dipeptide-binding ABC transporter, periplasmic substrate-binding component was detected in the genome of strain M86.It has been found that all the clinical isolates and the type strains of *Ochrobactrum* were highly resistant to all forms of ß-lactams except imipenem [16]. This resistance profile is consistent with the expression of the *AmpC* beta-lactamase characterized in *O. anthropi*[17,18]. The genome of strain M86 shows the presence of *AmpC* beta-lactamase gene which supports its resistance to ß-lactams antibiotic observed by Dharne et al. [4] .Table 1 shows the comparison of genomic features of *Ochrobactrum intermedium* strain M86 and *Ochrobactrum intermedium* LMG 3301^T^.

**Table 1:** **Comparisons of subsystem features between genome of *Ochrobactrum intermedium *strain M86 and *Ochrobactrum intermedium *LMG 3301**^**T**^

**Subsystem features**	**Number of CDS present in *O. intermedium***	
	**LMG 3301^T^**	**M86**
Amino acids and derivatives	448	464
Carbohydrates	460	483
Cofactors, vitamins, prosthetic groups, pigments	251	259
Protein metabolism	202	207
Membrane transport	188	225
Respiration	146	152
RNA Metabolism	131	130
DNA Metabolism	133	151
Cell wall and capsule	129	124
Stress response	121	126
Fatty acids, lipids, and isoprenoids	116	118
Nucleosides and nucleotides	105	105
Virulence, disease and defense	83	94
Motility and Chemotaxis	84	82
Regulation and cell signaling	66	72
Iron acquisition and metabolism	53	50
Miscellaneous	58	60
Phosphorus metabolism	46	44
Metabolism of aromatic compounds	29	30
Nitrogen metabolism	25	25
Cell division and cell cycle	17	17
Potassium metabolism	15	16
Sulfur metabolism	28	31

### Prediction of phage islands

The presence of prophage sequences may also allow some bacteria to acquire antibiotic resistance, to exist in new environmental niches, to improve adhesion or to become pathogenic [14]. In addition, phages play a crucial role in genome plasticity and chromosome remodelling. The intact phage of 32 kb in length together with phage-like proteins and hypothetical proteins were identified in the genome of strain M86 by prophage-predicting PHAST [14] Web server.

## Future directions

The genomic properties of *O. intermedium* are poorly characterized and, as a consequence, their role in human health and disease remains unclear. Elucidation of the physiological properties and identification of genes putatively involved in the various metabolic pathways may lead to a better understanding of the survival of *O. intermdedium* in acidic condition. Further studies involving large scale genome sequencing and comparison of the *O. intermedium* strains isolated from several other non-ulcer dyspeptic individuals will help us apprehend the genomic features of its survival in the acidic condition of the stomach. Comparative genomic analyses of *O. intemedium* strain M86 and other environmental isolates of genus *Ochrobactrum* will permit us to understand its mechanisms for adaptation to new environments.

## Competing interests

The authors declare that they have no competing interests.

## Authors’ contributions

GK and DD were involved in the genome assembly, annotation and manuscript preparation. SC and SS generated ion PGM data. MD and MP were involved in isolation of M86 strain. SM and VM provided the human stomach biopsy. GK and YS were involved in overall experimental design. All authors have read the manuscript and approved.

## References

[B1] Velasco J Romero C Lopez-Goni I Leiva J Diaz R Moriyon I Evaluation of the relatedness of *Brucella* spp. and *Ochrobactrum anthropi* and description of *Ochrobactrum intermedium* sp. nov., a new species with a closer relationship to *Brucella* spp Int J Syst Bacteriol 1998 48 759 768 10.1099/00207713-48-3-759 9734029

[B2] Moller LV Arends JP Harmsen HJ Talens A Terpstra PM Slooff J *Ochrobactrum intermedium* infection after liver transplantation J Clin Microbiol 1999 37 241 244 9854103 10.1128/jcm.37.1.241-244.1999 PMC84223

[B3] Apisarnthanarak A Kiratisin P Mundy LM Evaluation of *Ochrobactrum intermedium* bacteremia in a patient with bladder cancer Diagn Microbiol Infect Dis 2005 53 153 155 10.1016/j.diagmicrobio.2005.05.014 16168615

[B4] Dharne MS Misra SP Misra V Dwivedi M Patole MS Shouche YS Isolation of urease-positive *Ochrobactrum intermedium* in the stomach of a non-ulcer dyspeptic patient from north India J Microbiol Immunol Infect 2008 41 183 186 18473108

[B5] Khanolkar-Gaitonde SS Reubish GK Lee CK Stadtlander CT Isolation of bacteria other than *Helicobacter pylori* from stomachs of squirrel monkeys (*Saimiri* spp.) with gastritis Dig Dis Sci 2000 45 272 80 10.1023/A:1005496022881 10711437

[B6] Heilmann KL Borchard F Gastritis due to spiral shaped bacteria other than *Helicobacter pylori*: clinical, histological, and ultrastructural findings Gut 1991 32 137 140 10.1136/gut.32.2.137 1864530 PMC1378794

[B7] Hala MT El-Zimaty Ramchatesingh J Clarrige JE Abudayyeh S Osato MS Graham D Enterococcal gastritis Human Pathol 2003 34 944 945 10.1016/S0046-8177(03)00287-9 14562292

[B8] Brandi G Biavati B Calabrese C Granata M Nannetti A Mattarelli P Di Febo G Saccoccio G Biasco G Urease-positive bacteria other than *Helicobacter pylori* in human gastric juice and mucosa Am J Gastroenterol 2006 101 1756 61 10.1111/j.1572-0241.2006.00698.x 16780553

[B9] Sung L Changsung K Young C Successful cultivation of a potentially pathogenic coccoid organism with tropism for gastric mucin Infect Immun 1997 65 49 52 8975891 10.1128/iai.65.1.49-54.1997 PMC174555

[B10] Chevreux B Wetter T Suhai S Genome sequence assembly using trace signals and additional sequence information Comput Sci Biol 1999 99 45 56

[B11] Aziz RK Bartels D Best AA DeJongh M Disz T Edwards RA Formsma K Gerdes S Glass EM Kubal M Meyer F Olsen GJ Olson R Osterman AL Overbeek RA McNeil LK Paarmann D Paczian T Parrello B Pusch GD Reich C Stevens R Vassieva O Vonstein V Wilke A Zagnitko O The RAST Server: rapid annotations using subsystems technology BMC Genomics 2008 8 9 75 10.1186/1471-2164-9-75 PMC2265698 18261238

[B12] Hemmerich C Buechlein A Podicheti R Revanna KV Dong Q An Ergatis-based prokaryotic genome annotation web server Bioinformatics 2010 26 1122 1124 10.1093/bioinformatics/btq090 20194626

[B13] Delcher AL Harmon D Kasif S White O Salzberg SL Improved microbial gene identification with GLIMMER Nucleic Acids Res 1999 27 4636 4641 10.1093/nar/27.23.4636 10556321 PMC148753

[B14] Zhou Y Liang Y Lynch KH Dennis JJ Wishart DS PHAST: a fast phage search tool Nucleic Acids Res 2011 39 347 352 10.1093/nar/gkq749 21672955 PMC3125810

[B15] Kusters JG van Vliet AH Kuipers EJ Pathogenesis of *Helicobacter pylori* infection Clin Microbiol Rev 2006 19 449 490 10.1128/CMR.00054-05 16847081 PMC1539101

[B16] Teyssier C Marchandin H Jean-Pierre H Darbas H Siméon De Buochberg M Diego I Gouby A Jumas-Bilak E Molecular and phenotypic features for identification of the opportunistic pathogens *Ochrobactrum* spp J Med Microbiol 2005 54 945 953 10.1099/jmm.0.46116-0 16157548

[B17] Higgins CS Avison MB Jamieson L Simm AM Bennett PM Walsh TR Characterization, cloning and sequence analysis of the inducible *Ochrobactrum anthropi* AmpC ß-lactamase J Antimicrob Chemother 2001 47 745 754 10.1093/jac/47.6.745 11389106

[B18] Nadjar D Labia R Cerceau C Bizet C Philippon A Arlet G Molecular characterization of chromosomal class C ß-lactamase and its regulatory gene in *Ochrobactrum anthropi* Antimicrob Agents Chemother 2001 45 2324 2330 10.1128/AAC.45.8.2324-2330.2001 11451692 PMC90649

